# Potentiated Response of ERK/MAPK Signaling is Associated with Prolonged Withdrawal from Cocaine Behavioral Sensitization

**DOI:** 10.1007/s12031-021-01799-6

**Published:** 2021-01-21

**Authors:** Alexey Bingor, Matityahu Azriel, Lavi Amiad, Rami Yaka

**Affiliations:** Institute for Drug Research (IDR), School of Pharmacy, Faculty of Medicine, The Hebrew University of Jerusalem, 91120 Jerusalem, Israel

**Keywords:** Cocaine, ERK, NMDA, NR2B, Behavioral sensitization, Ifenprodil

## Abstract

Among the neuroadaptations underlying the expression of cocaine-induced behaviors are modifications in glutamate-mediated signaling and synaptic plasticity via activation of mitogen-activated protein kinases (MAPKs) within the nucleus accumbens (NAc). We hypothesized that exposure to cocaine leads to alterations in MAPK signaling in NAc neurons, which facilitates changes in the glutamatergic system and thus behavioral changes. We have previously shown that following withdrawal from cocaine-induced behavioral sensitization (BS), an increase in glutamate receptor expression and elevated MAPK signaling was evident. Here, we set out to determine the time course and behavioral consequences of inhibition of extracellular signal-regulated kinase (ERK) or NMDA receptors following withdrawal from BS. We found that inhibiting ERK by microinjection of U0126 into the NAc at 1 or 6 days following withdrawal from BS did not affect the expression of BS when challenged with cocaine at 14 days. However, inhibition of ERK 1 day before the cocaine challenge abolished the expression of BS. We also inhibited NR2B-containing NMDA receptors in the NAc by microinjection of ifenprodil into the NAc following withdrawal from BS, which had no effect on the expression of BS. However, microinjection of ifenprodil to the NAc 1 day before challenge attenuated the expression of BS similar to ERK inhibition. These results suggest that following a prolonged period of withdrawal, NR2B-containing NMDA receptors and ERK activity play a critical role in the expression of cocaine behavioral sensitization.

## Introduction

Repeated injections of psychoactive drugs, such as cocaine, induce a behavioral response termed behavioral sensitization (BS). BS reflects neuronal adaptations that lead to drug seeking and craving by augmenting the incentive, motivational value of psychoactive drugs (Kalivas and Stewart 1991; Robinson 1993). One of the major neuronal adaptations of the brain’s reward circuitry involves modifications in glutamate receptor function and signaling in the ventral tegmental area (VTA) and nucleus accumbens (NAc) (Wolf 1998; Kalivas 2004). Blocking NMDA receptor function in the VTA by its specific antagonist MK-801 abolished the initiation of cocaine-mediated BS (Kalivas and Alesdatter 1993), stressing the importance of NMDA receptors in controlling dopaminergic neuron activity in the VTA.

The long-term molecular and synaptic modifications in the NAc induced by exposure to cocaine following withdrawal have been studied extensively (Malenka et al. 2007). Among these changes are alterations in glutamatergic receptor function and subsequent signaling through activation of extracellular signal-regulated kinase (ERK)-mediated pathways (Girault et al. 2007; Thomas et al. 2008). Cocaine BS increases the membrane expression of the GluA1 subunit of the AMPA receptor (AMPAR-GluA1) in the NAc at 21 days, but not 1 day, after withdrawal from cocaine (Boudreau and Wolf 2005). In line with this study, electrophysiological experiments determining the ratio of AMPA/NMDA receptor-mediated currents (in vivo long-term potentiation (LTP)) demonstrated that in cocaine-sensitized mice 2 weeks following the last injection, increased AMPA/NMDA was observed in NAc slices, suggesting that prolonged withdrawal from repeated cocaine administration increases synaptic strength in the NAc neurons (Kourrich et al. 2007).

In addition, increased surface expression of AMPAR-GluA1 correlated with an increase in ERK activity in cocaine-sensitized rats (Boudreau et al. 2007). Phosphorylation and activation of ERK is known to mediate the behavioral effects of cocaine (Lu et al. 2006; Girault et al. 2007), including BS (Berhow et al. 1996; Valjent et al. 2004; Mattson et al. 2005). Inhibition of ERK signaling by the MEK antagonist PD98059 injection into the VTA, prior to repeated cocaine injections, attenuated cocaine-induced behavioral sensitization (Pierce et al. 1999). Likewise, systemic delivery of MEK inhibitor SL327 prior to repeated cocaine injections interfered with the development phase of cocaine BS (Valjent et al. 2006). Finally, in rats sensitized with cocaine, increases in ERK activation and phosphorylation of cAMP response element-binding protein (CREB) were blocked by infusions of the MEK inhibitor U0126 into the NAc (Mattson et al. 2005). These results reflect the pivotal role of ERK signaling pathway in the neuronal processes responsible for the development and expression of cocaine behavioral sensitization.

We have previously shown that repeated noncontingent cocaine injections increased the expression of NMDA receptor subunits NR1, NR2A, NR2B, and GluA1 in the NAc at 3 weeks, but not 1 day, after withdrawal from cocaine (Schumann and Yaka 2009). We also found a time-dependent increase in ERK activity that correlated with the increased expression of NMDA receptor subunits. Furthermore, blocking NR2B-containing NMDA receptor function by ifenprodil during the development phase of sensitization, negatively affect the expression of BS. Finally, inhibition of ERK by the MEK inhibitor U0126 in the NAc 3 weeks following withdrawal from cocaine resulted in inhibition of cocaine-induced increase in the expression of GluA1. In the current study, we aimed to examine the time course of direct ERK inhibition or NMDA receptor antagonism in the NAc during withdrawal from BS and the effect of such inhibition on the expression of sensitization.

## Materials and Methods

### Animals

The Hebrew University strain of Sabra rats were housed with food and water available ad libitum, a standard 12-h light/dark cycle (7 am–7 pm) in groups of four (Harlan Laboratories, Jerusalem). Injections and behavioral sessions were done on male rats 20–35 days old between 10:00 am and 4:00 pm. The Institutional Animal Care and Use Committee of the Hebrew University of Jerusalem approved all of the procedures described.

### Behavioral Sensitization

Rats were assigned to saline and cocaine treatment groups after a week of acclimation to their homecage environment. One day before the first cocaine or saline injection, rats were habituated to the behavioral testing procedure by placement in photocell cages (MED Associates; St. Albans, VT) for 30 min after saline injection. On the first day of treatment (day 1), rats were habituated to photocell cages for 20 min before injection of cocaine (15 mg/kg, i.p.) or saline (1 ml/kg, i.p.). Locomotor activity (total beam breaks) was measured for an additional 30 min. For the next 4 days (days 2–5), the same procedure was applied. Criteria for sensitization were based on the coefficient of variance (CV) as described previously (Boudreau and Wolf 2005). After day 5, cocaine injections were withdrawn until a single challenge dose (15 mg/kg) provided at day 18; locomotor activity was again determined over 30 min.

### Surgery and Microinjection

Following anesthesia, rats were implanted with 28 gauge guide cannulae (small parts), which were placed bilaterally 1.7 mm anterior to bregma, ± 2.0 mm mediolateral, 4.6 mm ventral to the skull surface corresponding to 2 mm above the NAc (Paxinos and Watson 2007). Behavioral sensitization was performed 7 days following implantation. Injection cannulae were inserted to extend 2 mm beyond the end of the guide cannulae and drugs infused into the NAc over 2 min at a pump-controlled rate of 0.25 μl per minute. The injection needle was kept in place for an additional 2 min following cessation of infusion to ensure adequate diffusion from the needle tip. ERK inhibitor U0126 solution (Calbiochem, 0.5 μl injection volume, 1 μg/μl in 5% DMSO, 6% Tween 80 in PBS (Carnicella et al. 2008)) or vehicle was infused into the NAc at the time points indicated. Similarly, the NR2B antagonist ifenprodil (Sigma), or the appropriate vehicle, was microinjected into the NAc (0.5 μl injection volume, 1 μg/μl in sterile saline 0.9%).

### Western Blot Analysis

Protein (50 µg) from brain homogenates was resolved by 10% SDS-PAGE and transferred to a nitrocellulose membrane. The membranes were incubated overnight at 4 °C with appropriate primary antibodies and then incubated (1 h at room temperature) with appropriate IRDye conjugated fluorescent secondary antibodies (Rockland Immunochemicals). IRDye conjugates are optimized for the Odyssey Infrared Imaging System (LI-COR Biosciences). The bands were quantitatively analyzed by densitometry, with National Institutes of Health Image providing peak areas, and values were expressed as the percentage of control. Anti-pERK and anti-ERK antibodies were from Cell Signaling Technology.

### Statistical Analysis

Pairwise comparisons were performed on behavioral test data, using an independent *t* test. To analyze effects of cocaine and each inhibitor, two-way ANOVA was performed and significant interactions determined by Bonferroni post hoc test to correct for multiple comparisons. Data were considered significant at *p* values of < 0.05.

## Results

### Inhibition of ERK in the NAc Following Cocaine Withdrawal Can Prevent the Expression of BS Only Prior to Cocaine Challenge

**Fig. 1:**
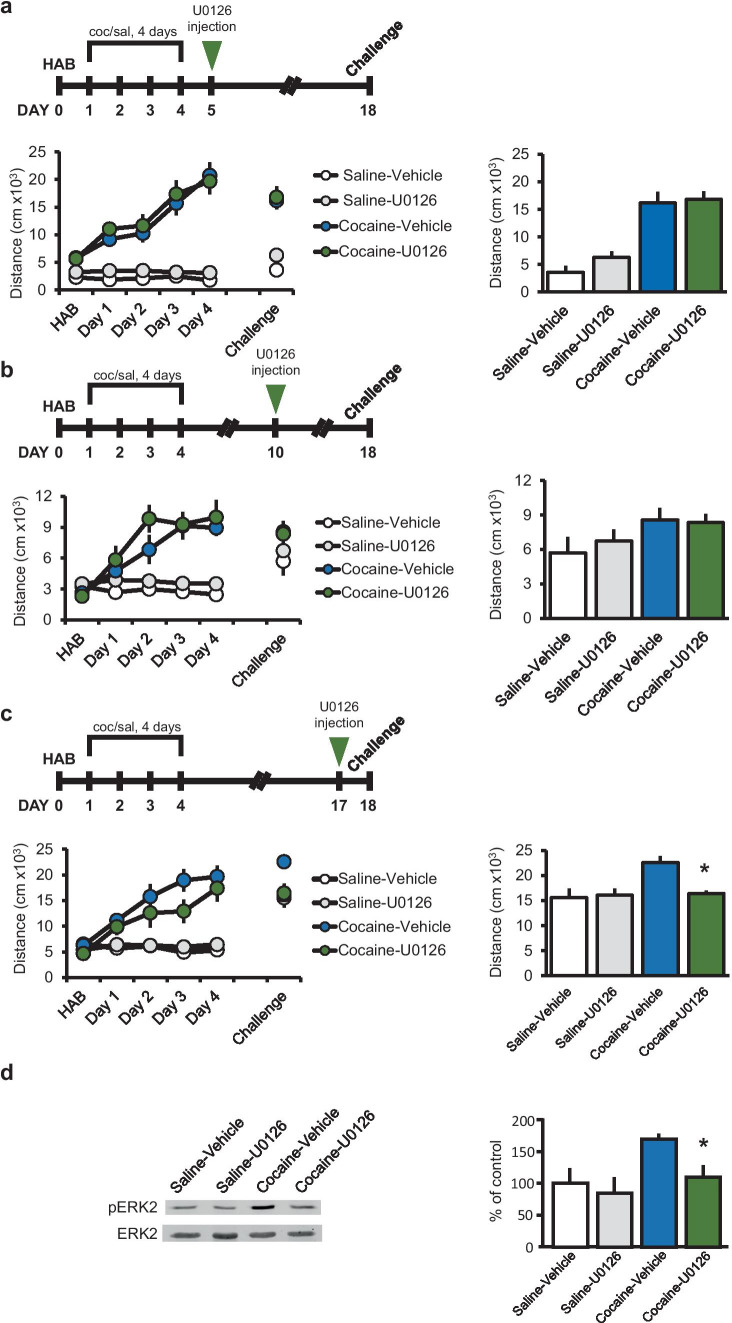
Inhibition of ERK in the NAc by the MEK inhibitor U0126 13 days (but not 1 or 6 days) following withdrawal decreases the expression of BS. **a** Rats were habituated and sensitized to cocaine in a behavioral sensitization (BS) procedure, as described in the “Materials and Methods” section. One day after repeated injections of cocaine (15 mg/kg) or saline, both cocaine- and saline-treated rats were microinjected bilaterally with U0126 (a total of 0.5 μl of U0126 at 1 μg/μl) or with vehicle (vehicle, 0.5 μl per side) into the NAc. Rats were exposed to a cocaine challenge 14 days after last cocaine injection. No significant difference in the distance was observed between cocaine + U0126-treated (*N* = 10) vs cocaine + vehicle treated (*N* = 10) groups or between saline + U0126 treated (*N* = 8) vs saline + vehicle treated (*N* = 8) groups. **b** Rats were sensitized to cocaine as described in **a**, injected with U0126 or vehicle 6 days after the training period and exposed to a cocaine challenge 14 days after last cocaine injection. No significant difference in distance was observed between cocaine + U0126 treated (*N* = 8) vs cocaine + vehicle treated (*N* = 8) groups. **c** Rats were sensitized to cocaine as described in **a**, injected with U0126 or vehicle 13 days after the training period and exposed to challenge procedure one day after injection of the inhibitor. A significant decrease (*p < 0.05) in distance from cocaine + U0126-treated group (*N* = 8) was observed when compared to cocaine + vehicle-treated group (*N* = 8; two-way ANOVA, followed by Bonferroni post hoc test). No significant difference in distance traveled from saline + U0126-treated (*N* = 8) vs saline + vehicle-treated (*N* = 8) groups. **d** Rats were treated as in **c**. Rats were immediately killed and NAc tissue blocks were dissected, and the levels of pERK2 and ERK2 were detected using specific antibodies. Histograms depict the level of ERK2 phosphorylation (phospho-p42) normalized to total ERK (total p42) from five independent experiments. Data are presented as mean ± SEM percentage of saline-vehicle. Significant difference was found between cocaine-vehicle vs cocaine-U0126; *p < 0.01

We have previously shown that prolonged withdrawal from noncontingent cocaine injections is correlated with time-dependent gradual increase in ERK activity in the NAc of rats (Schumann and Yaka 2009). However, the effect of ERK inhibition on the expression of BS following subsequent cocaine challenge remains unknown. Therefore, we directly injected the MEK inhibitor U1026 into the NAc at different time points following cocaine training determine at what stage ERK inhibition will attenuate the expression of BS. U0126 was initially microinjected into the NAc one day following repeated cocaine injections (day 5), at a concentration (2.3 mM) known to inhibit ERK activity (Schumann and Yaka 2009; Duncia et al. 1998; London and Clayton 2008). Rats were subsequently challenged with cocaine at day 18, and locomotor activity did not differ from vehicle-injected animals (Fig. 1a). We previously observed increased pERK at 1 week following cessation of injections (Schumann and Yaka 2009). Therefore, we examined the effect of ERK inhibition at this time point and found that U0126-mediated inhibition of ERK at day 10 did not affect locomotor activity following cocaine challenge injection at day 18 (Fig. 1b). Finally, we examined whether inhibition of ERK 1 day before challenge would affect the expression of BS. Rats were microinjected with U0126 to the NAc on day 17 before challenge with cocaine on day 18. Inhibiting ERK resulted in a significant decrease in cocaine-induced locomotor activity, to the same level as for rats that had received repeated saline injections (Fig. 1c). To ensure that ERK activity is inhibited by U0126, rats were sacrificed on day 18 following challenge injection and the levels of ERK phosphorylation was determined. As shown in Fig. 1d, in cocaine-treated rats, we found a significant increase of ERK phosphorylation; however, in rats that were microinjected with U0126 into the NAc, ERK phosphorylation returned to baseline similar to rats treated with U0126 alone or saline control animals. Together, these results suggest that although ERK activation is evident 7 days after withdrawal from repeated cocaine injections (Schumann and Yaka 2009), inhibition of ERK can prevent the expression of BS only when given a day prior to cocaine challenge.

### Inhibition of NR2B-Containing NMDA Receptors in the NAc During Withdrawal from Repeated Cocaine Injections Did Not Affect the Expression of BS

**Fig. 2:**
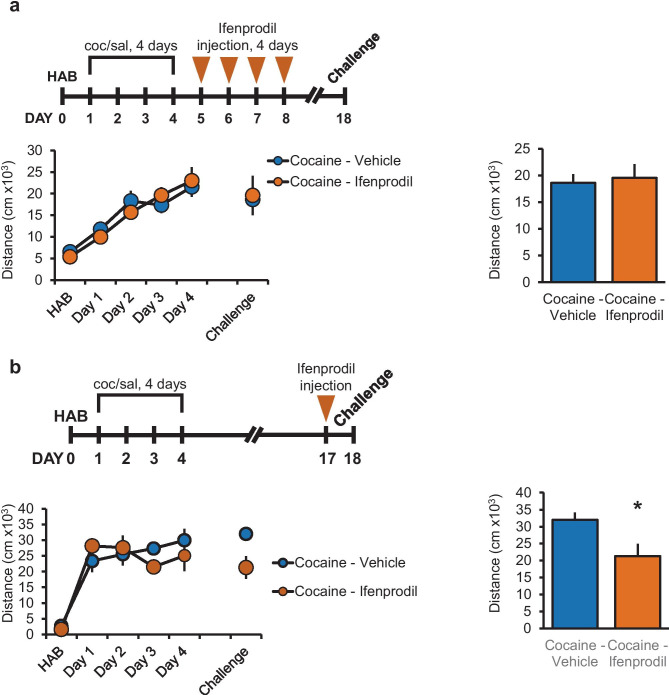
Inhibition of NR2B-containing NMDAR receptor in the NAc by ifenprodil 13 days (but not at first 4 days) following withdrawal decreases the expression of BSA. Rats were sensitized to cocaine as in Fig. 1a. From the first day after last cocaine injections, animals were microinjected with ifenprodil (0.5 μg/side) into the NAc for 4 consecutive days. Rats were exposed to challenge procedure 14 days after last cocaine injection. No significant difference in distance traveled was observed between cocaine + ifenprodil-treated (*N* = 8) vs cocaine + vehicle-treated (*N* = 8) groups. **b** Cocaine-sensitized rats were injected with ifenprodil into the NAc 13 days after the training period and exposed to challenge procedure 1 day after injection of the inhibitor. A significant decrease (*p < 0.05; one-tailed unpaired *t* test) in distance from cocaine + ifenprodil-treated group (*N* = 6) was observed when compared to cocaine + vehicle-treated group (*N* = 4)

Prolonged withdrawal from BS results in an increase in NR1, NR2A, and NR2B subunit expression in the NAc of rats (Schumann and Yaka 2009). We also showed that inhibiting NR2B-containing NMDA receptors by systemic injections of ifenprodil, prior to each cocaine injection during the development of BS, significantly attenuated cocaine BS. In the current study, we aimed to evaluate the contribution of NR2B-containing NMDA receptors in NAc to the expression of BS. We therefore microinjected ifenprodil into the NAc, 1 day following repeated cocaine injections (day 5) and continuously for the next 3 days, at a concentration (3.1 mM) known to inhibit NR2B-NMDA receptors (Gitto et al. 2014), and on day 18, rats were challenged with single cocaine injection. Inhibition of NR2B-containing NMDA receptors in the NAc during withdrawal from repeated cocaine injections did not prevent the expression of BS, and the levels of locomotor activity did not differ from vehicle-injected group (Fig. 2a).

### Inhibition of NR2B-Containing NMDA Receptors in the NAc After 13 days of Withdrawal Inhibits Expression of BS After Subsequent Challenge

Since NMDA receptors are upstream of ERK activation (Schumann and Yaka 2009), we hypothesized that, like ERK inhibition prior to challenge injection with cocaine, inhibition of NR2B-containing NMDA receptor will result in attenuation in the expression of BS. Therefore, ifenprodil was microinjected into the NAc at day 17 before challenge with cocaine on day 18. Indeed, ifenprodil decreased the expression of BS tested 1 day following injection (Fig. 2b). These results suggest that while chronic inhibition of NR2B-containing NMDA receptors in the NAc during the first days of withdrawal did not affect the expression of BS, acute inhibition of NR2B-containing NMDA receptors in the NAc after 13 days of withdrawal and 1 day before challenge negatively affected the expression of BS.

## Discussion

The results of the current study indicate that withdrawal from repeated noncontingent cocaine injections induces time-dependent alterations in ERK activation, which are responsible for the expression of behavioral sensitization (BS) at late stages of withdrawal. We examined the time course of direct ERK inhibition (U0126) or blockade of NR2B-containing NMDA receptor (ifenprodil) in the NAc during withdrawal from BS. U0126 inhibits MEK1 and MEK2 with IC_50_ of 0.07 μM and 0.06 μM respectively (Duncia et al. 1998) and has a half-life of 2 h (London and Clayton 2008), sufficient for our experiments. We found that inhibiting ERK one day before a challenge injection (day 13 of withdrawal) of cocaine, but not 1 or 7 days after withdrawal, prevented the expression of BS. Similarly, we show that while chronic inhibition of NR2B-containing NMDA receptors by microinjection of ifenprodil into the NAc at early stage of withdrawal did not affect the expression of cocaine sensitization, inhibition of these receptors in the NAc at later stage of withdrawal (1 day before challenge) decreased the expression of BS. These findings suggest that activation of ERK and NR2B-containing NMDA receptors following prolonged withdrawal is critical to manifest the expression of cocaine-induced sensitization.

The role of the MAPK family member ERK, its intracellular signaling and subsequent neuroadaptive changes, have been studied intensely (Sun et al. 2016). ERK activation is considered to be critical for associative learning (Atkins et al. 1998). Amplification in ERK signaling results both in persistent changes in structural plasticity, such as increased dendritic spine density (Ren et al. 2010) and in synaptic plasticity, such as increased AMPA receptors density on neuronal synaptic membranes (Zhu et al. 2002). In particular, the effects of the most commonly used drugs of abuse including cocaine, or their active components, on ERK signaling in the principal areas of the reward system has been extensively studied (Zhai et al. 2008). ERK is expressed throughout the brain (Ortiz et al. 1995) and is highly expressed within the dopaminergic reward system, emphasizing the important role of ERK signaling in modulating the activity of the reward system under normal function and following exposure to drugs of abuse. ERK signaling has therefore been studied in paradigms of drug dependence, withdrawal and relapse. ERK plays a role in cocaine-induced behaviors (Lu et al. 2005, 2006; Girault et al. 2007), including behavioral sensitization (Berhow et al. 1996; Valjent et al. 2004; Mattson et al. 2005; Girault et al. 2007). The development of cocaine behavioral sensitization was attenuated by inhibiting ERK activation, which also abolished cocaine-conditioned reward following CPP (Pierce et al. 1999; Valjent et al. 2000, 2006; Miller and Marshall 2005). Presentation of cocaine-associated cues during self-administration to cocaine enhances ERK phosphorylation in the central amygdala at 30 days but not 1 day after withdrawal from cocaine. Additionally, inhibition of ERK phosphorylation under the same behavioral procedure attenuates the number of lever presses for cocaine-associated cues (Lu et al. 2005). In line with these experiments, we also found that inhibition of ERK at an early stage following BS did not affect the expression of BS; its impact on BS was apparent only when ERK was inhibited 2 weeks later, a day prior to challenge. We suggest that withdrawal from cocaine administration induces the activation of ERK, regardless of the presentation of environmental cues. These results also suggest that although ERK activation is sustained during withdrawal following BS (Schumann and Yaka 2009), ERK signaling in the NAc is more sensitive for interference at late withdrawal periods to prevent the reinstatement of cocaine sensitization.

It was previously shown that inhibition of ERK by intraperitoneal (i.p.) injection had no effect on the expression of sensitization, i.e., it did not decrease the enhanced locomotor response when it was administered just before the challenge injection (Valjent et al. 2005). However, this study used a different MEK inhibitor—SL327. Moreover, i.p. injection depends on the pharmacokinetics and distribution of this drug to the brain and specifically to the NAc, while microinjection directly to the NAc ensure that the compound does not depends on the methodology of delivery. In addition, in this study, SL327 was injected shortly before challenge injection while in the current study U0126 was microinjected 1 day before challenge. We suggest that inhibition of the ERK-induced increase of GluA1 into the synaptic membrane is insufficient to block the expression of sensitization, when inhibitor is delivered right before challenge, particularly when the inhibitor is administered systemically.

We have previously demonstrated that inhibition of NMDA receptors containing the NR2B subunit by systemic injection of ifenprodil during repeated injections of cocaine (sensitization) decreases the development and expression of BS (Schumann and Yaka 2009). We have also shown that 1-day following withdrawal, synaptic NMDA receptor subunits NR1, NR2A, and NR2B were decreased and internalized but 3 weeks after withdrawal there was a significant increase in both the expression and synaptic localization of these subunits. In the current study, we hypothesized that chronic inhibition of NR2B-containing NMDA receptors during the first days of withdrawal will negatively affect the expression of BS in NAc-dependent manner. However, we found no change in the expression of BS following chronic intra-accumbens inhibition of NR2B-containing receptors during the early phase of withdrawal. In contrast, acute inhibition of NR2B-containing receptors in the NAc after 13 days of withdrawal (when NMDA receptor expression was elevated (Schumann and Yaka 2009)), and 1 day before challenge, attenuated the expression of cocaine-BS. These results may suggest that the NMDA receptor-mediated signaling during withdrawal from repeated cocaine injections is not critical for the expression of BS, at least in the first days following withdrawal. These results also correlate with the lack of effect shown following ERK inhibition during the early period of withdrawal. It is also possible that inhibition of ERK or NR2B during withdrawal does not prevent the expression of BS due to the timing of injections; chronic inhibition throughout the withdrawal period (not necessarily at early stages of withdrawal) may be required. However, it seems that both NR2B-NMDA receptor and its downstream target MAPK are critical for the expression of BS since independent inhibition of both proteins before cocaine challenge attenuated the expression of BS.

Taken together, we suggest that increased glutamate signaling developed in the NAc during withdrawal from repeated cocaine exposure via NR2B-containing NMDA receptors is responsible for the increase in ERK activity. Increased ERK activation leads to an enhanced expression of AMPA-GluA1 receptors in the synaptic membrane, and amplified AMPA receptor-mediated glutamate transmission is responsible for the expression of BS, tested by challenge injection. Therefore, blocking the NR2B-NMDA receptor or its downstream MAPK pathway in cocaine-sensitized rats will prevent the expression of BS. This proposed mechanism is in line with previous studies demonstrating that AMPA receptors lacking GluA2, (presumably compensated by increased AMPA-GluA1 receptors), are responsible for increased sensitivity of NAc neurons for drug associated cues in the incubation of cocaine craving paradigm (Conrad et al. 2008; Dong et al. 2017).

Our results indicate that the NMDA receptor-ERK pathway is critical for the establishment of long-term alterations of behavioral responses to a second exposure to cocaine. We suggest that the ERK signaling cascade is a hub of convergence of signaling pathways triggered following withdrawal from drug administration. ERK signaling may potentially be a target for therapeutic intervention to prevent the expression of drug-induced behaviors at late withdrawal periods.

## Data Availability

The data used to support the findings of this study are available from the corresponding author upon request.

## References

[CR1] Atkins CM, Selcher JC, Petraitis JJ, Trzaskos JM, Sweatt JD (1998). The MAPK cascade is required for mammalian associative learning. Nat Neurosci.

[CR2] Berhow MT, Hiroi N, Nestler EJ (1996). Regulation of ERK (extracellular signal regulated kinase), part of the neurotrophin signal transduction cascade, in the rat mesolimbic dopamine system by chronic exposure to morphine or cocaine. J Neurosci.

[CR3] Boudreau AC, Reimers JM, Milovanovic M, Wolf ME (2007). Cell surface AMPA receptors in the rat nucleus accumbens increase during cocaine withdrawal but internalize after cocaine challenge in association with altered activation of mitogen-activated protein kinases. J Neurosci.

[CR4] Boudreau AC, Wolf ME (2005). Behavioral sensitization to cocaine is associated with increased AMPA receptor surface expression in the nucleus accumbens. J Neurosci.

[CR5] Carnicella S, Kharazia V, Jeanblanc J, Janak PH, Ron D (2008). GDNF is a fast-acting potent inhibitor of alcohol consumption and relapse. Proc Natl Acad Sci U S A.

[CR6] Conrad KL, Tseng KY, Uejima JL, Reimers JM, Heng L-J, Shaham Y (2008). Formation of accumbens GluR2-lacking AMPA receptors mediates incubation of cocaine craving. Nature.

[CR7] Dong Y, Taylor JR, Wolf ME, Shaham Y (2017). Circuit and synaptic plasticity mechanisms of drug relapse. J Neurosci.

[CR8] Duncia JV, Santella JB, Higley CA, Pitts WJ, Wityak J, Frietze WE (1998). MEK inhibitors: the chemistry and biological activity of U0126, its analogs, and cyclization products. Bioorg Med Chem Lett.

[CR9] Girault J, Valjent E, Caboche J, Herve D (2007). ERK2: a logical AND gate critical for drug-induced plasticity?. Curr Opin Pharmacol.

[CR10] Gitto R, De Luca L, Ferro S, Russo E, De Sarro G, Chisari M (2014). Synthesis, modelling and biological characterization of 3-substituted-1H-indoles as ligands of GluN2B-containing N-methyl-d-aspartate receptors. Bioorg Med Chem.

[CR11] Kalivas P (2004). Glutamate systems in cocaine addiction. Curr Opin Pharmacol.

[CR12] Kalivas, P. W., and Alesdatter, J. E. (1993). Involvement of N-methyl-D-aspartate receptor stimulation in the ventral tegmental area and amygdala in behavioral sensitization to cocaine. J. Pharmacol. Exp. Ther. 267, 486–95. Available at: http://www.ncbi.nlm.nih.gov/pubmed/8229779. 8229779

[CR13] Kalivas PW, Stewart J (1991). Dopamine transmission in the initiation and expression of drug- and stress-induced sensitization of motor activity. Brain Res Rev.

[CR14] Kourrich S, Rothwell PE, Klug JR, Thomas MJ (2007). Cocaine experience controls bidirectional synaptic plasticity in the nucleus accumbens. J Neurosci.

[CR16] London SE, Clayton DF (2008). Functional identification of sensory mechanisms required for developmental song learning. Nat Neurosci.

[CR17] Lu L, Hope BT, Dempsey J, Liu SY, Bossert JM, Shaham Y (2005). Central amygdala ERK signaling pathway is critical to incubation of cocaine craving. Nat Neurosci.

[CR18] Lu L, Koya E, Zhai H, Hope BT, Shaham Y (2006). Role of ERK in cocaine addiction. Trends Neurosci.

[CR19] Malenka RC, Kauer J, a, Malenka, R. C., and Kauer, J. a,  (2007). Synaptic plasticity and addiction. Nat Rev Neurosci.

[CR20] Mattson BJ, Bossert JM, Simmons DE, Nozaki N, Nagarkar D, Kreuter JD (2005). Cocaine-induced CREB phosphorylation in nucleus accumbens of cocaine-sensitized rats is enabled by enhanced activation of extracellular signal-related kinase, but not protein kinase A. J Neurochem.

[CR21] Miller CA, Marshall JF (2005). Molecular substrates for retrieval and reconsolidation of cocaine-associated contextual memory. Neuron.

[CR22] Ortiz, J., Harris, H. W., Guitart, X., Terwilliger, R. Z., Haycock, J. W., and Nestler, E. J. (1995). Extracellular signal-regulated protein kinases (ERKs) and ERK kinase (MEK) in brain: regional distribution and regulation by chronic morphine. J. Neurosci. 15, 1285–97. Available at: http://www.ncbi.nlm.nih.gov/pubmed/7532701. 10.1523/JNEUROSCI.15-02-01285.1995 PMC6577831 7532701

[CR23] Paxinos, G., and Watson, C. (2007). The Rat Brain in Stereotaxic Coordinates Sixth Edition. 10.1016/0165-0270(80)90021-7 6110810

[CR24] Pierce RC, Pierce-Bancroft AF, Prasad BM (1999). Neurotrophin-3 contributes to the initiation of behavioral sensitization to cocaine by activating the Ras/Mitogen-activated protein kinase signal transduction cascade. J Neurosci.

[CR26] Ren Z, Sun WL, Jiao H, Zhang D, Kong H, Wang X (2010). Dopamine D1 and N-methyl-d-aspartate receptors and extracellular signal-regulated kinase mediate neuronal morphological changes induced by repeated cocaine administration. Neuroscience.

[CR27] Robinson T (1993). The neural basis of drug craving: an incentive-sensitization theory of addiction. Brain Res Rev.

[CR30] Schumann J, Yaka R (2009). Prolonged withdrawal from repeated noncontingent cocaine exposure increases NMDA receptor expression and ERK activity in the nucleus accumbens. J Neurosci.

[CR32] Sun, W.-L., Quizon, P. M., and Zhu, J. (2016). “Molecular Mechanism: ERK Signaling, Drug Addiction, and Behavioral Effects,” in, 1–40. 10.1016/bs.pmbts.2015.10.017. 10.1016/bs.pmbts.2015.10.017 PMC5330621 26809997

[CR33] Thomas MJ, Kalivas PW, Shaham Y (2008). Neuroplasticity in the mesolimbic dopamine system and cocaine addiction. Br J Pharmacol.

[CR34] Valjent E, Corvol J-C, Pagès C, Besson M-J, Maldonado R, Caboche J (2000). Involvement of the extracellular signal-regulated kinase cascade for cocaine-rewarding properties. J Neurosci.

[CR35] Valjent E, Corvol J-C, Trzaskos JM, Girault J-A, Hervé D (2006). Role of the ERK pathway in psychostimulant-induced locomotor sensitization. BMC Neurosci.

[CR36] Valjent E, Pages C, Herve D, Girault JA, Caboche J (2004). Addictive and non-addictive drugs induce distinct and specific patterns of ERK activation in mouse brain. Eur J Neurosci.

[CR37] Valjent E, Pascoli V, Svenningsson P, Paul S, Enslen H, Corvol J-C (2005). From the cover: regulation of a protein phosphatase cascade allows convergent dopamine and glutamate signals to activate ERK in the striatum. Proc Natl Acad Sci.

[CR38] Wolf ME (1998). The role of excitatory amino acids in behavioral sensitization to psychomotor stimulants. Prog Neurobiol.

[CR39] Zhai H, Li Y, Wang X, Lu L (2008). Drug-induced alterations in the extracellular signal-regulated kinase (ERK) signalling pathway: implications for reinforcement and reinstatement. Cell Mol Neurobiol.

[CR40] Zhu JJ, Qin Y, Zhao M, Van Aelst L, Malinow R (2002). Ras and Rap control AMPA receptor trafficking during synaptic plasticity. Cell.

